# Supplementary material for: Triple A patient cells suffering from mitotic defects fail to localize PGRMC1 to mitotic kinetochore fibers
Source: Cell Div. 2018 Nov 10;13:8. doi: 10.1186/s13008-018-0041-5 (PMC6230297; doi:10.1186/s13008-018-0041-5)
Supplement: Supplementary file 1 — Additional file 1. Figure S1: Multi-level regression models of Figure 1A. Figure S2: Multi-level regression models of Figure 1B. Figure S3: Full-length western blot of Figure 4B. Figure S4: TaqMan analysis of PGRMC1 in human skin fibroblasts. Table S1: Real-time qPCR primer oligonucleotides. [file 13008_2018_41_MOESM1_ESM.pdf]

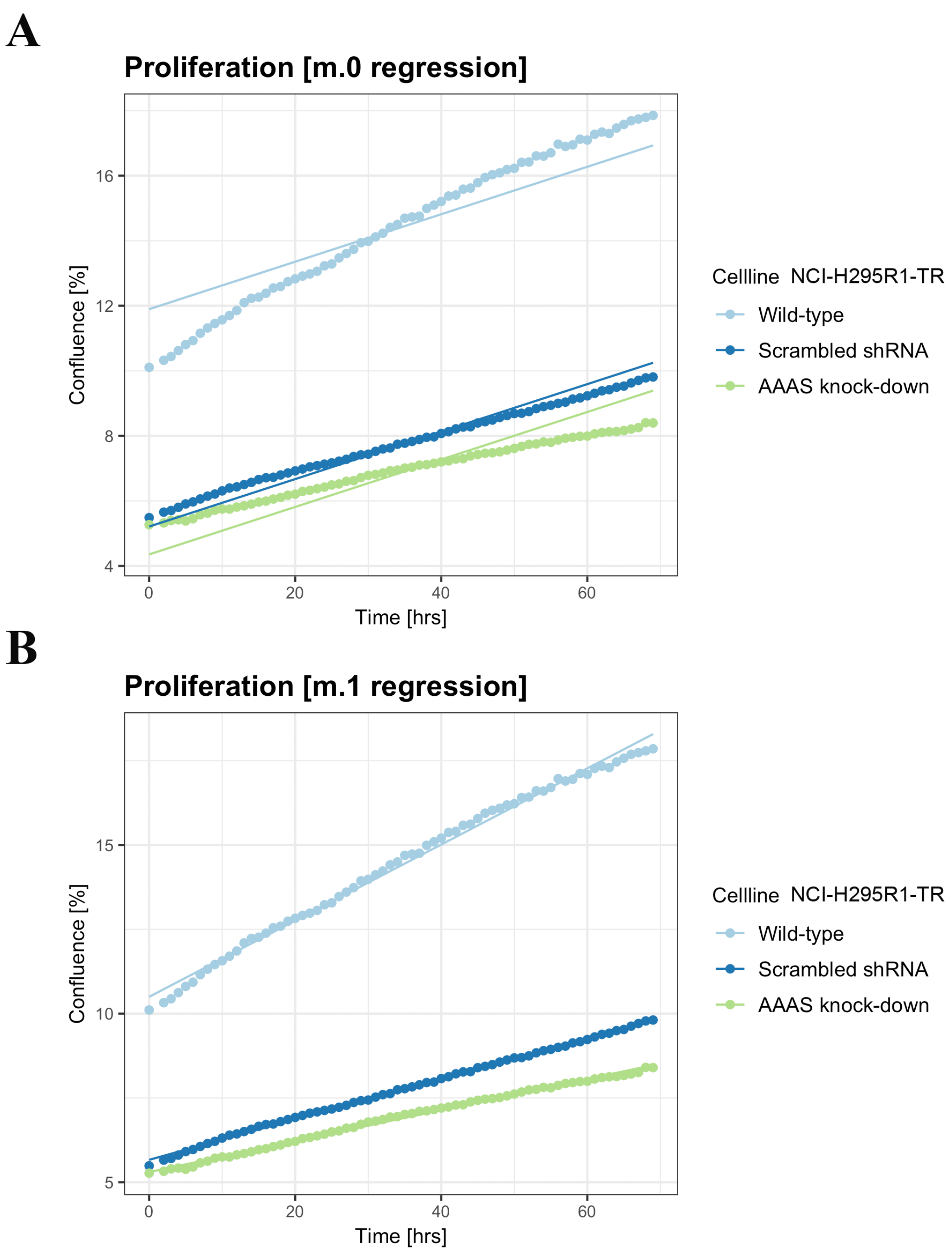

Figure S1 - Multi-level regression models of Figure 1A.

Growth curve analysis (GCA) was done using multilevel regression modeling using R Studio as described earlier [58, 59]. In **A**) the intercept model (m.0) and in **B**) the linear model (m.1) is presented. The intercept model suggests constant differences in proliferation randomly assigned to the different cell lines and the linear model suggests effects of the different cell lines on proliferation. Statistical comparison (Pearson's chi-squared test) of these two models revealed that the linear model (m.1) is our regression model of choice ($p < 0.001$).

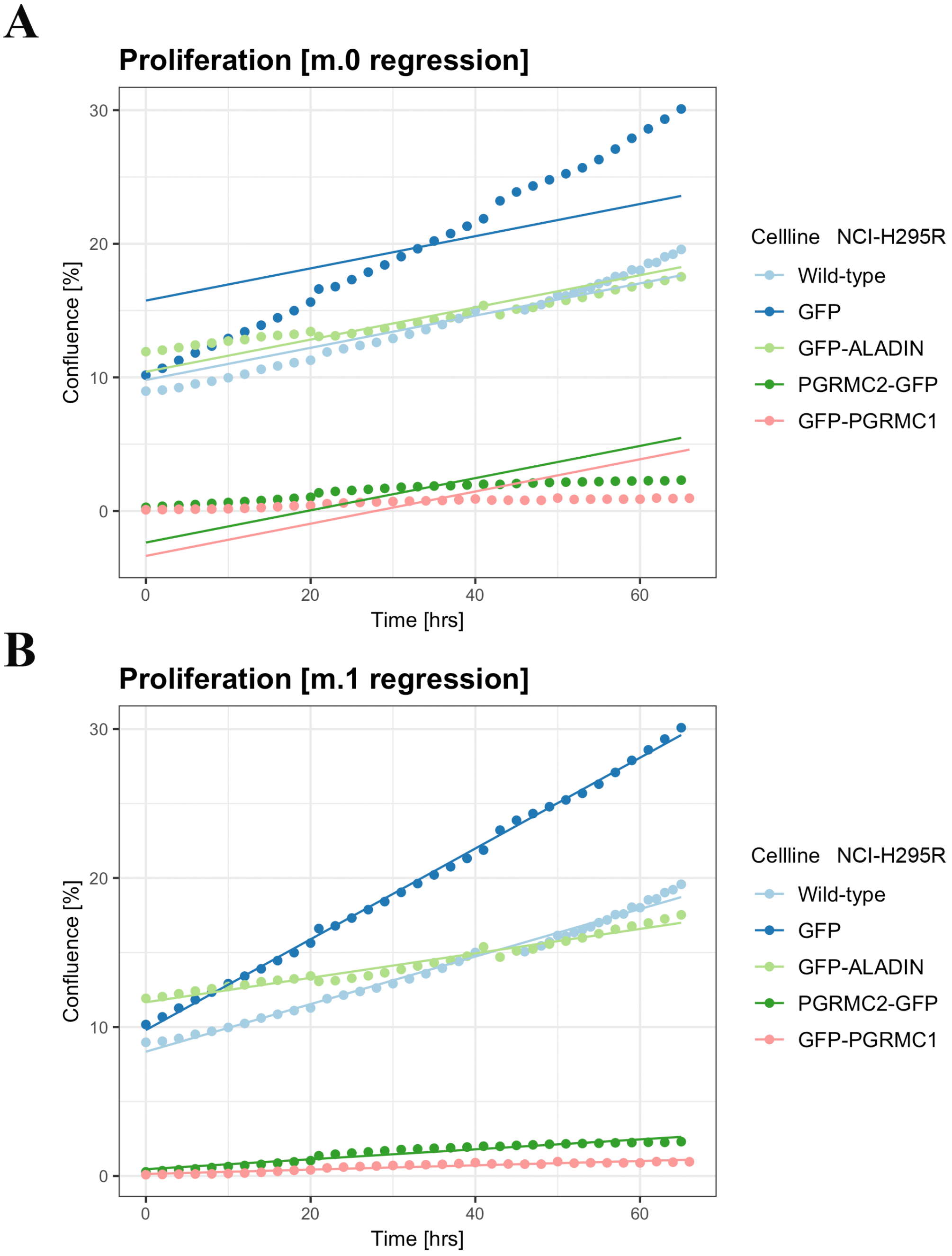

Figure S2 - Multi-level regression models of Figure 1B.

Growth curve analysis (GCA) was done using multilevel regression modeling using R Studio as described earlier [58, 59]. In **A**) the intercept model (m.0) and in **B**) the linear model (m.1) is presented. The intercept model suggests constant differences in proliferation randomly assigned to the different cell lines and the linear model suggests effects of the different cell lines on proliferation. Statistical comparison (Pearson's chi-squared test) of these two models revealed that the linear model (m.1) is our regression model of choice ($p < 0.001$).

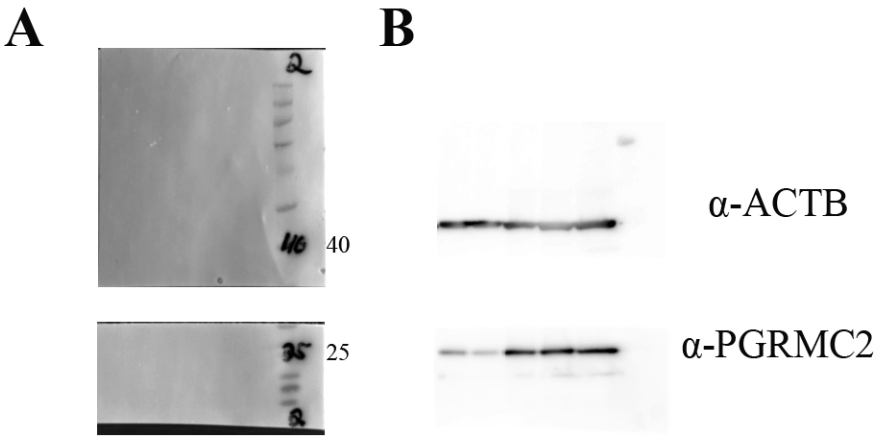

Figure S3 - Full-length western blot of Figure 4B.
Unprocessed full-length western blot of Figure 4B. In **A**) the piece of nitrocellulose membrane is shown along with the molecular weight marker and in **B**) the original image acquired with Azure Imager is presented.

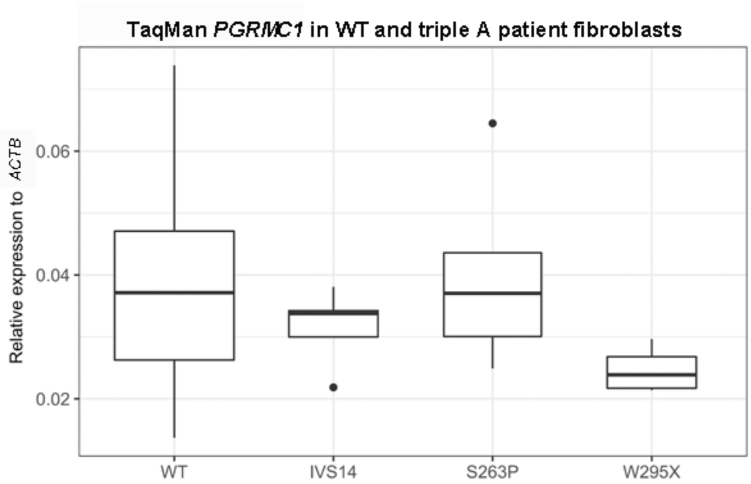

Figure S4 - TaqMan analysis of *PGRMC1* in human skin fibroblasts.
Total RNA was isolated from human skin fibroblasts of healthy wild-type donors and triple A patients. The different mutations in the human *AAAS* gene are denoted on the x-axis of the diagram: IVS14, S263P and W295X. WT, wild-type.

Table S1 - Real-time qPCR primer oligonucleotides.

GENE	NAME	SEQUENCE
ACTB (V1: NM_001101.3)	*ACTB*-F	GCACCCAGCACAATGAAGATC
	ACTB-R	CGCAACTAAGTCATAGTCCGC
	ACTB-Probe	TGCTCCTCCTGAGCGCAAGTACTCC
PGRMC1 (V1: NM_006667.4)	*PGRMC1*-F	CTGCATGATTTCTGTTTTATCTACCTCTA
	PGRMC1-R	TGTTACTGGACAGCGCTTAATCC
	PGRMC1-Probe	AGCAAATCTGCAGTGTTCCAAAGACTTTGG
PGRMC2 (V1: NM 006320.4)	*PGRMC2*-F	GCGGTCAATGGGAAAGTCTTC
	PGRMC2-R	CTACCAGCAAATATTCCATA
	PGRMC2-Probe	AGTTCTACGGCCCCGCGGGTC
